# Simple approach for the fabrication of PEDOT-coated Si nanowires

**DOI:** 10.3762/bjnano.6.65

**Published:** 2015-03-04

**Authors:** Mingxuan Zhu, Marielle Eyraud, Judikael Le Rouzo, Nadia Ait Ahmed, Florence Boulc’h, Claude Alfonso, Philippe Knauth, François Flory

**Affiliations:** 1 Aix-Marseille University, Institut Matériaux Microélectronique Nanosciences de Provence-IM2NP, CNRS-UMR 7334, équipe OPTO-PV, Domaine Universitaire de Saint-Jérôme, Service 231, 13397 Marseille Cedex 20, France; 2 Ecole Centrale Marseille, 38 rue Joliot Curie, 13451 Marseille Cedex 20, France; 3 Shanghai Institute of Technical Physics, Chinese Academy of Sciences, 500 Yutian Road, 200240 Shanghai, China; 4 Aix-Marseille University, CNRS, MADIREL UMR 7246, équipe Electrochimie des Matériaux, 13397 Marseille Cedex 20, France,; 5 Université Abderrahmane Mira, Lab. d’Electrochimie, Corrosion et de Valorisation énergétique, 06000 Bejaia, Algeria

**Keywords:** conductive polymer, core–shell structure, electrodeposition, hybrid material, SiNW

## Abstract

The synthesis of a conformal poly(3,4-ethylenedioxythiophene) (PEDOT) layer on Si nanowires was demonstrated using a pulsed electrodeposition technique. N-type Si nanowire (SiNWs) arrays were synthesized using an electroless metal-assisted chemical etching technique. The dependence of the SiNW reflection on the concentration of the AgNO_3_ solution was identified. A reflection of less than 2% over the entire visible spectral range was obtained for these structures, evidencing their excellent antireflective properties. The etched SiNWs nanostructures can be further modified by using a tapering technique, which further preserves the strong light trapping effect. P-type PEDOT was grown on these SiNWs using electrochemical methods. Since the polymerization reaction is a very fast process with regards to monomer diffusion along the SiNW, the conformal deposition by classical, fixed potential deposition was not favored. Instead, the core–shell heterojunction structure was finally achieved by a pulsed deposition method. An extremely large shunt resistance was exhibited and determined to be related to the diffusion conditions occurring during polymerization.

## Introduction

Silicon nanowires (SiNWs) are a current, active research topic for many applications such as photovoltaics [1], lithium batteries [2], hydrogen storage [3] and optoelectronic devices [4] due to their unique properties with respect to visible light management [5–7]. Using an electroless etching method, a reflectivity as low as 1.3% over the entire visible spectrum can be achieved for SiNWs [8]. As far as the device fabrication is concerned, a core–shell arrangement of p–n junction forming materials is promising for the optimization of the electronic charge collection capability. This is due to the nature of the core–shell structure, which allows the transport path along the radial direction of photogenerated carriers to be greatly shortened without sacrificing light absorption [9].

However, the high aspect ratio of SiNWs makes it difficult to realize a radial p–n junction, where each individual Si wire in the array would need to be individually coated. Various fabrication efforts have been attempted to achieve a true core–shell p–n junction. For example, chemical vapor deposition (CVD) [10–11] and atomic layer deposition (ALD) [12] are methods that can be employed to obtain this type of nanostructured junction, however, they suffer from high cost. The combination of spin-on doping (SOD) and rapid thermal annealing (RTM) was also attempted to achieve a core–shell Si homojunction [13–14], but this method failed to precisely control the thickness of the shell. Core–shell, radial p–n junctions can also be realized by simply spin coating poly(3,4-ethylenedioxythiophene)/polystyrene sulfonate (PEDOT/PSS) (a successful, commercial, conducting polymer) onto a SiNW array. This gave very promising results for photovoltaic cells based on this heterojunction, with a photon capture efficiency (PCE) of 6.72% [15]. The resulting SiNW/PEDOT/PSS heterojunction showed rectification behavior with a large saturation current density. A relatively low shunt resistance and a high saturation current are displayed by devices produced using the spin coating method. This is because the PEDOT:PSS only partially covers the SiNWs array, leaving most of the SiNW surface uncovered [16]. In order to improve the junction quality, a conformal PEDOT shell should be introduced to eliminate charge transport paths parallel to the diode.

Compared with the spin coating technique, the electrochemical polymerization of PEDOT provides the possibility of excellent polymer morphology control by tuning the thickness and reaction rate. Template polymerization of PEDOT has already been investigated on nanostructures such as ZnO [17], TiO_2_ [18], GaAs [19], AAO [20]. However, using a SiNW array as template for PEDOT deposition is an unexplored research field with only a few existing publications [21–22]. Some relevant questions are: (i) What is the role of nanostructured Si in PEDOT nucleation and growth? (ii) How do the deposition conditions influence the PEDOT properties? (iii) How can the diode quality be improved?

In this paper, we will first present the influence of the AgNO_3_ concentration on the antireflection properties of SiNWs that were etched with the electroless metal-assisted chemical etching (EMACE) method. To determine the optimum electrodeposition parameters, a preliminary study was performed for 3,4-ethylenedioxythiophene (EDOT) oxidation on vitreous carbon (a conductive, non-electroactive, and easily-polished substrate). This was followed by EDOT oxidation on SiNW substrates. Optical and electrical properties, as well as morphology and composition of the samples, were determined using spectroscopy, current density–voltage curves, scanning electron and transmission electron microcopies, energy-dispersive X-ray analysis, and IR spectroscopy.

## Results and Discussion

### Effect of tapering on SiNW antireflection

**Figure 1:**
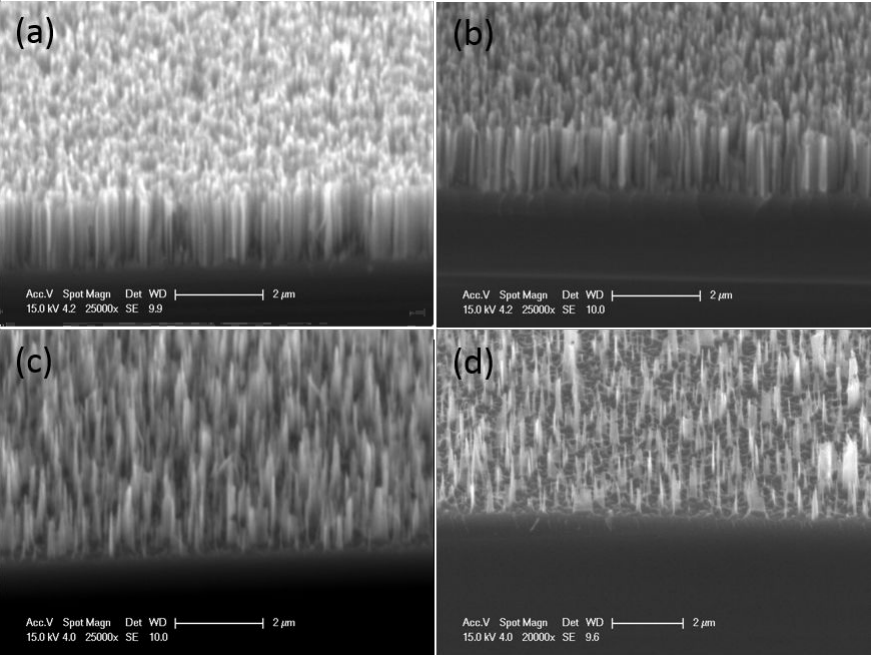
Tilted-view, SEM observations of SiNW samples without tapering (a) and for different tapering times: (b) 10 s, (c) 30 s, (d) 50 s.

**Figure 2:**
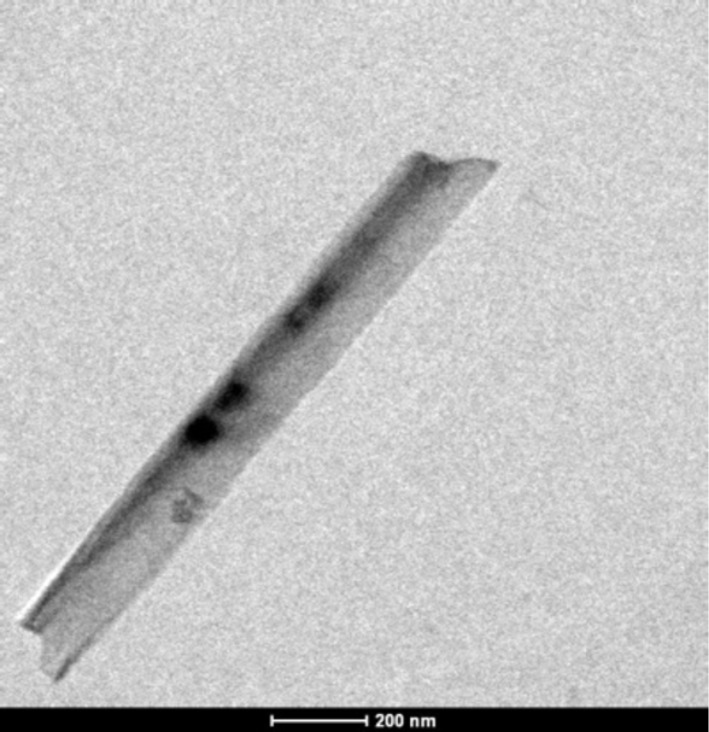
TEM image of a silicon nanowire obtained using the same conditions as those in Figure 1a.

The SiNWs, as prepared from an n-type Si substrate according to the process described in the Experimental section, can be seen in Figure 1. The geometry of the wires depends strongly on the experimental conditions and can be controlled. This work on SiNWs includes a detailed study of the dimensions of these structures as related to their properties. For instance, the length of the SiNW can be easily controlled by the etching duration and the concentration of the AgNO_3_ solution influences the morphology. However, for this study, only those SiNWs that exhibit good optical performance (low reflection at the surface, i.e., a light trapping effect) which were suitable for PEDOT deposition (using a KOH solution after the EMACE process) were addressed. The chosen experimental conditions resulted in a dense array of smooth Si nanowires, 2 µm in length, approximately 100 nm thick, and oriented perpendicular to the Si substrate. In this case, the space between the wires was quite small. TEM observation of the nanowires allows the dimensions to be measured more precisely. The polydispersity of the diameter is low with a diameter of 130 ± 5 nm (see Figure 2). The effect of the duration of the tapering step on the shape, length and density of the SiNWs is shown by comparing Figure 1a (without tapering) with Figure 1b–d, where the etching time increases from b to d. Clearly, by increasing the tapering duration, the space between wires is increased, the density of wires is reduced, and their top becomes sharper.

**Figure 3:**
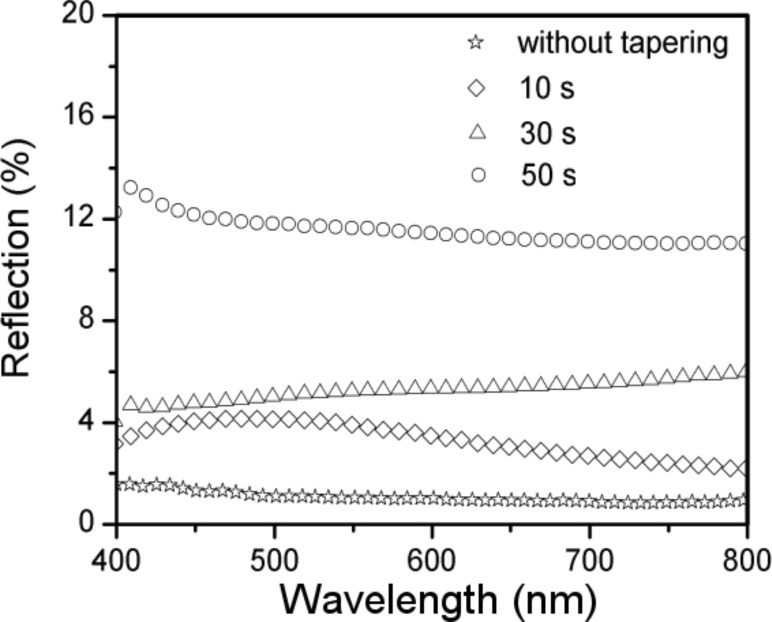
Reflection spectra of SiNWs in the visible spectral range without tapering and after 10, 30, and 50 s of tapering.

Spectrophotometric measurements were performed on these samples to determine their light absorption properties (Figure 3). Due to the strong light trapping effect [1], the SiNWs produced without tapering exhibit a reflection of less than 2% over the entire visible range. The reflection of the tapered samples increases with the tapering duration. After 50 s of tapering, the SiNWs have a reflection greater than 10% due to the reduced nanowire density on the substrate. For a tapering time of less than 30 s, the reflectance does not show significant change: a 10 s tapering leads to a reflection of less than 5% and a 30 s tapering to a reflection of less than 6%. This suggests that there is still a strong light trapping effect in the tapered SiNWs, although the morphology has been obviously modified. By tuning the interspace volume, the EDOT diffusion effect on the morphology of PEDOT can be further investigated, as will be discussed later.

### Cyclic voltammetry and FTIR analysis for PEDOT deposition on vitreous carbon

**Scheme 1:**
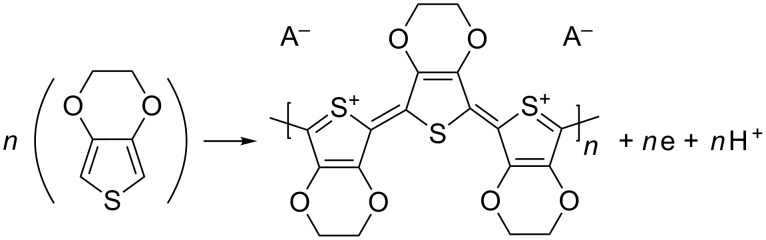
Polymerization reaction scheme for PEDOT synthesis.

**Figure 4:**
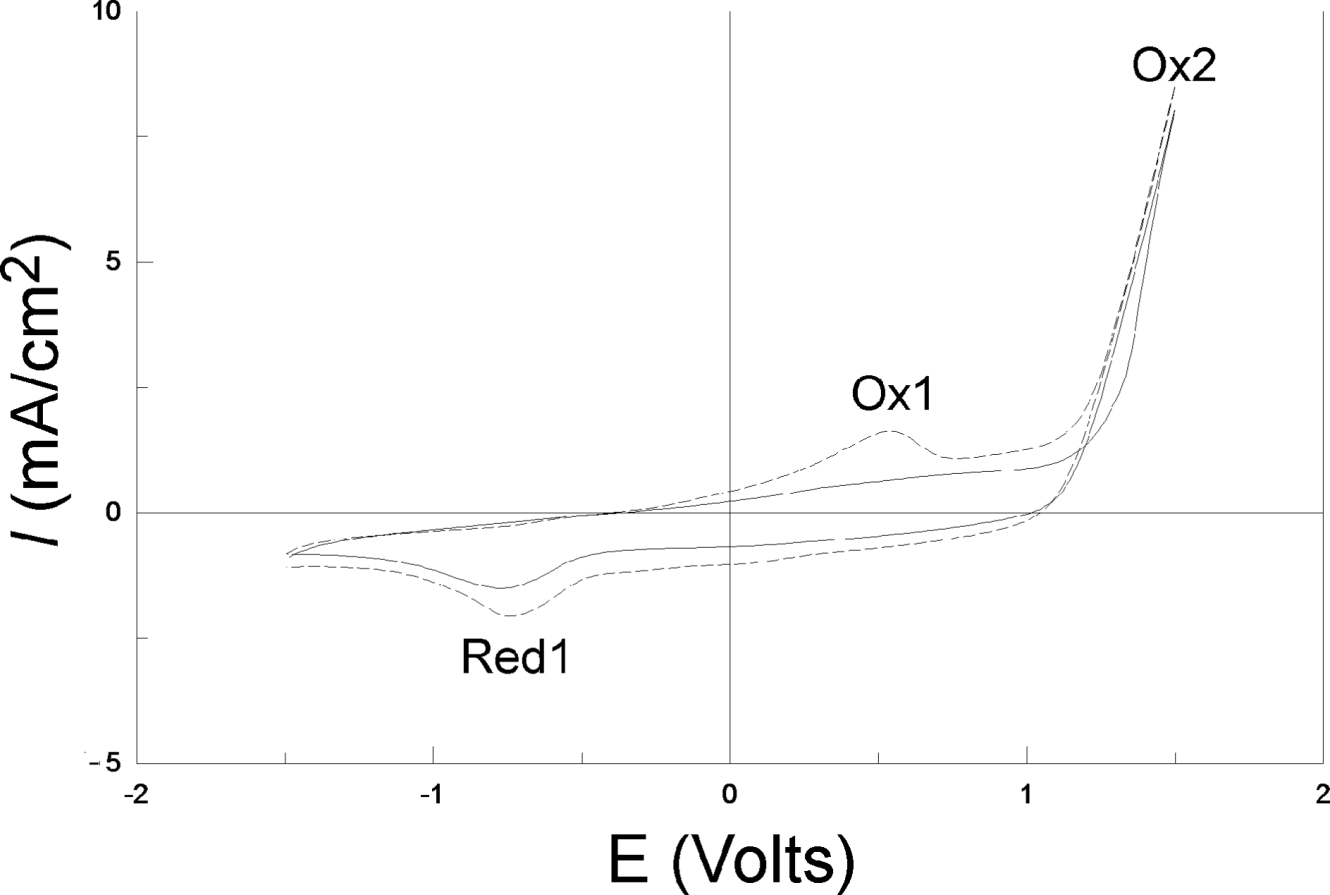
Two successive CVs performed on vitreous carbon with 10 mM EDOT and 0.1 M LiClO_4_ in acetonitrile solution. Scan rate: 100 mV/s. The solid line corresponds to the 1st scan and the dashed line to the 2nd scan.

Cyclic voltammetry (CV) experiments on vitreous carbon from EDOT containing solutions, starting from −1.5 to 1.5 V for 2 successive cycles, are shown in Figure 4. During the first scan (solid line), no current is observed up to a potential value of 1.3 V. Between 1.3 and 1.5 V, the first EDOT oxidation peak appears (see the Ox2 label in Figure 4), which is in good agreement with the literature [23–24]. This leads to the rapid synthesis of PEDOT according to the reaction in Scheme 1.

**Scheme 2:**
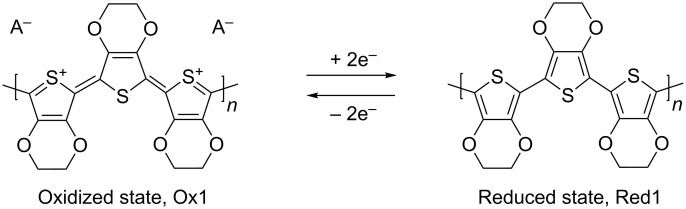
Polymer reduction reaction scheme for PEDOT.

When the potential is reversed, a crossover point between the forward and the backward current is observed between 1.3 and 1.1 V. This effect appears because it is easier to deposit PEDOT on PEDOT (during the reverse scan), than to deposit PEDOT on vitreous carbon (during the forward scan). This CV curve form is called a nucleation loop and implies that the PEDOT film completely covered the initial vitreous carbon surface. When the potential was decreased below 0.5 V, a small reduction peak (Red 1 in Figure 4) was exhibited during the reverse scan showing that the polymer can be reduced according to the reaction [24] in Scheme 2.

During the second scan (dashed line), a new oxidation peak (Ox1) appears around 0.5 V corresponding to the oxidization of PEDOT. The oxidation of EDOT into PEDOT near Ox2 appears at a lower potential than during the first scan (1.1 V), and no nucleation loop is observed. This is because the vitreous carbon substrate is completely covered by the PEDOT film during the first cycle. At the end of the experiment, the electrode is completely covered with a blue film.

**Figure 5:**
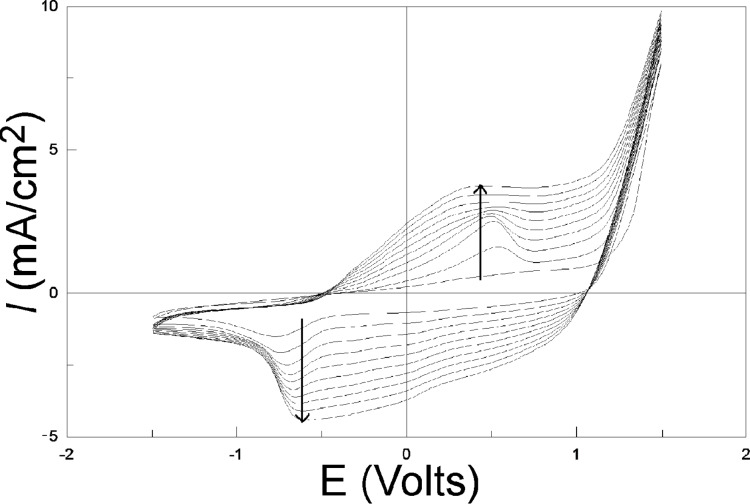
10 successive CVs performed on vitreous carbon with 10 mM EDOT and 0.1 M LiClO_4_ in acetonitrile solution. Scan rate: 100 mV/s. The arrow direction indicates increasing scan numbers.

Figure 5 presents 10 successive cycles performed on vitreous carbon. The increase of both the cathodic and anodic current densities for the Red1 (left arrow) and Ox1 (right arrow) peaks with increasing number of cycles is obvious, and is correlated with the thickening of the PEDOT film. On the contrary, the current at Ox2 due to the EDOT to PEDOT transformation is constant, indicating the good conductivity of the film.

**Figure 6:**
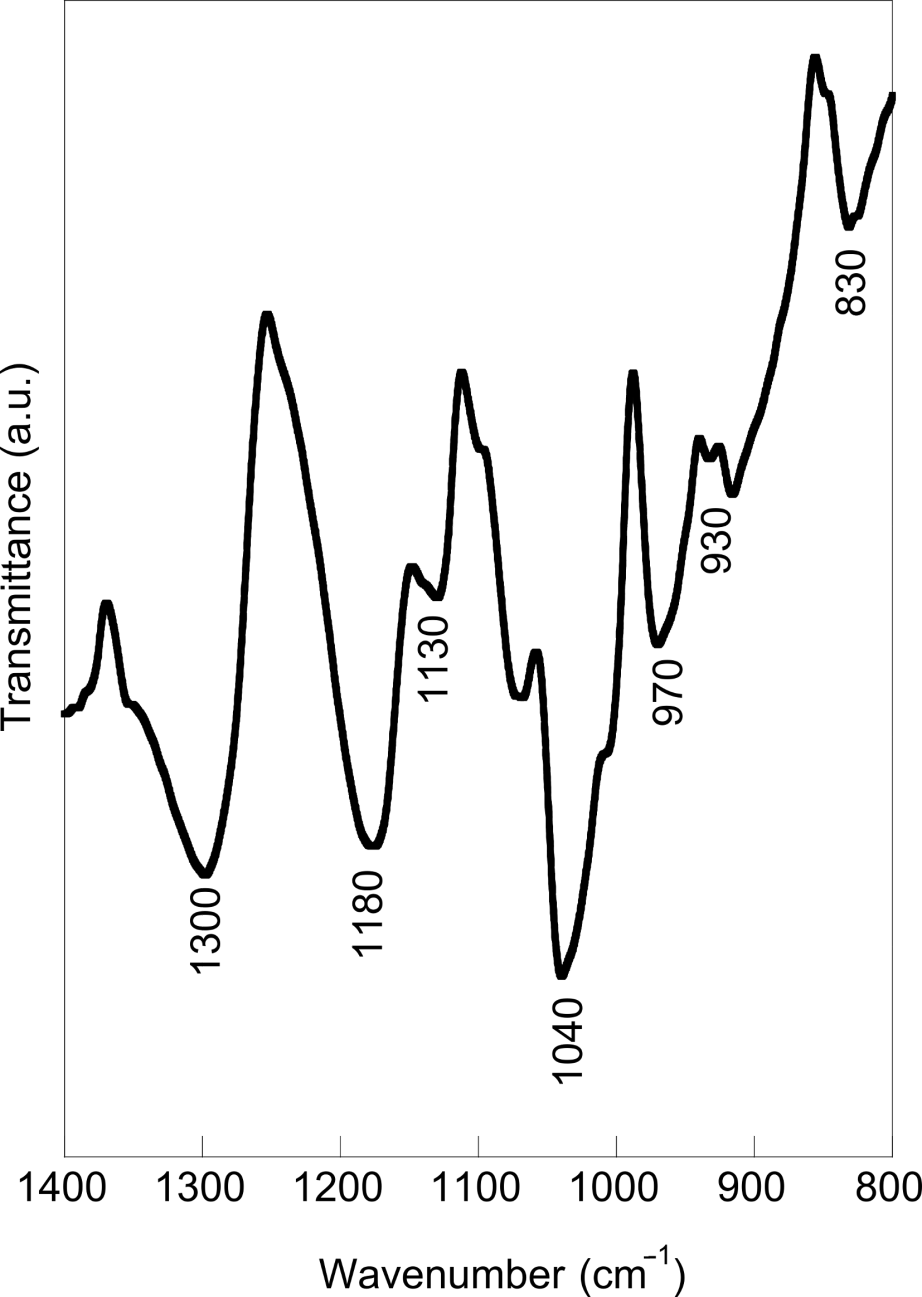
FTIR spectrum of a PEDOT film electropolymerized onto vitreous carbon.

To prove that a PEDOT film can be obtained under these conditions, a fixed voltage of 1.5 V was applied for 5 s in a 10 mM EDOT acetonotrile (ACN) solution with 0.1 M LiClO_4_ and the reflection FTIR spectra were recorded in the wavenumber region 1400–800 cm^−1^ (Figure 6). Additionally, Table 1 presents the principal assignments reported in literature for similar polythiophene films. Vibrations at 830, 930 and 970 cm^−1^ originate from the C–S bond in the thiophene ring [17,25–26]. Moreover, vibrations at 1040 cm^−1^, 1130 cm^−1^, 1180 cm^−1^ and 1300 cm^−1^ are assigned to stretching in the alkylenedioxy group [17,25–26]. These results confirm that the electrodeposited thin blue films are PEDOT.

**Table 1:** Proposed assignments for the main vibrations of polythiophene films.

Wavenumber (cm^−1^)	Assignment^a^	Reference

830 cm^−1^ 930 cm^−1^ 970 cm^−1^ 1040 cm^−1^ 1130 cm^−1^ 1180 cm^−1^ 1300 cm^−1^	C–S C–S C–S C=C / CO–R–OC CO–R–OC CO–R–OC CO–R–OC	[17,25–26] [25] [17] [25–26] [25–26] [25] [17]

^a^R represents CH_2_–CH_2_.

### Cyclic voltammetry of PEDOT deposition on SiNWs

**Figure 7:**
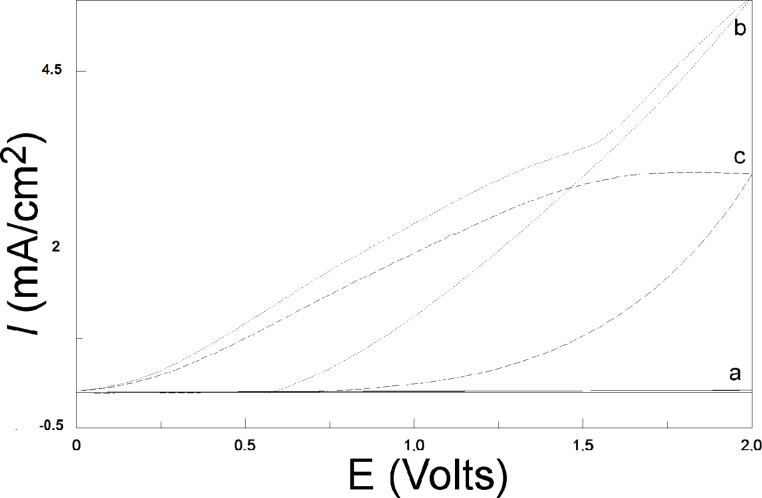
CVs of PEDOT deposition on SiNWs under various conditions. Scan rate: 100 mV/s. (a) 10 mM EDOT with 0.1 M LiClO_4_ in acetonitrile solution, without illumination; (b) 10 mM EDOT with 0.1 M LiClO_4_ in acetonitrile solution, under illumination; (c) 0.1 M LiClO_4_ in acetonitrile solution, under illumination.

Illumination by a concentrated light source is necessary for PEDOT polymerization on SiNWs electrodes. Indeed, the comparison of the CV curves in Figure 7 for EDOT under ambient light (curve a) shows that the current density is almost zero, much lower than that after illumination (curve b). The reason is that illumination may provide electron holes that increase the conductivity of the n-type SiNW substrate under anodic polarization. For all further experiments, the SiNW substrate was kept under constant illumination.

The CV measurement in an EDOT-containing solution (Figure 7, curve b) presents a characteristic inflection point at 1.3 V where the EDOT monomer begins to be oxidized and polymerized. After this inflection point, the current density in the EDOT-containing solution is much higher than that measured in the EDOT-free solution (curve c), suggesting that it arises from PEDOT polymerization. The first oxidation current that appears between 0 and 1.3 V in both EDOT-free (curve c) and EDOT-containng solutions (curve b) (absent in the glassy carbon substate, Figure 4) can be related to the oxidation of SiNWs to silica. The absence of the nucleation loop previously observed during the first cycle on vitreous carbon (Figure 4) is noted, and is likely due to this first large oxidation current.

**Figure 8:**
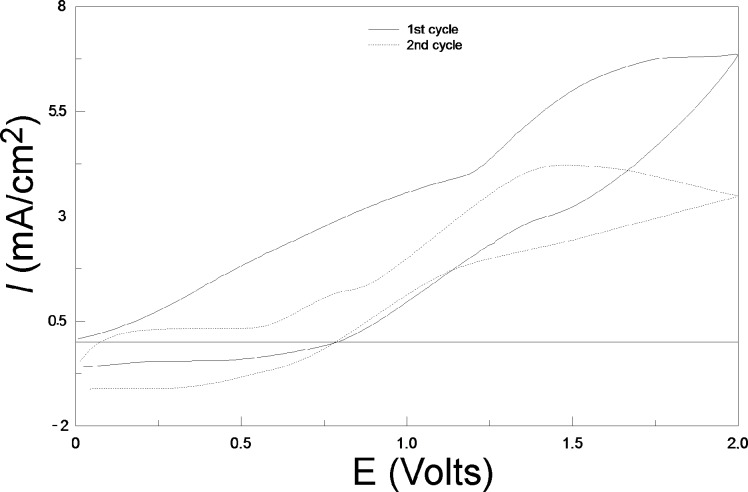
Two successive CVs performed on SiNWs under illumination with 10 mM EDOT and 0.1 M LiClO_4_ in acetonitrile solution. Scan rate: 100 mV/s.

Figure 8 presents two successive cycles corresponding to PEDOT deposition on SiNWs. Several notable differences appear between the two scans. First, the disappearance of the first large peak due to the oxidation of Si in SiO_2_ can be observed. This could happen because the PEDOT layer obtained during the first scan already covers the substrate. Also, the small peak around 0.5 V corresponds to the oxidation of the previously reduced PEDOT layer. It can also be observed that polymerization of EDOT in PEDOT is achieved at a lower anodic potential (0.9 V) than previously (1.3 V). Finally, a large decrease in the PEDOT formation current in comparison with that obtained during the first cycle can be observed, likely due to the presence of the insulating silica sublayer.

### PEDOT deposition on SiNWs using 1.5 V constant potential

For PEDOT deposition, a constant potential deposition was preferred over cycling in order to avoid a silica layer that was thicker than desired. Indeed, during CV deposition, the oxidation of Si in SiO_2_ was observed before the deposition of PEDOT, while at fixed potential, simultaneous Si and EDOT oxidation occurred.

**Figure 9:**
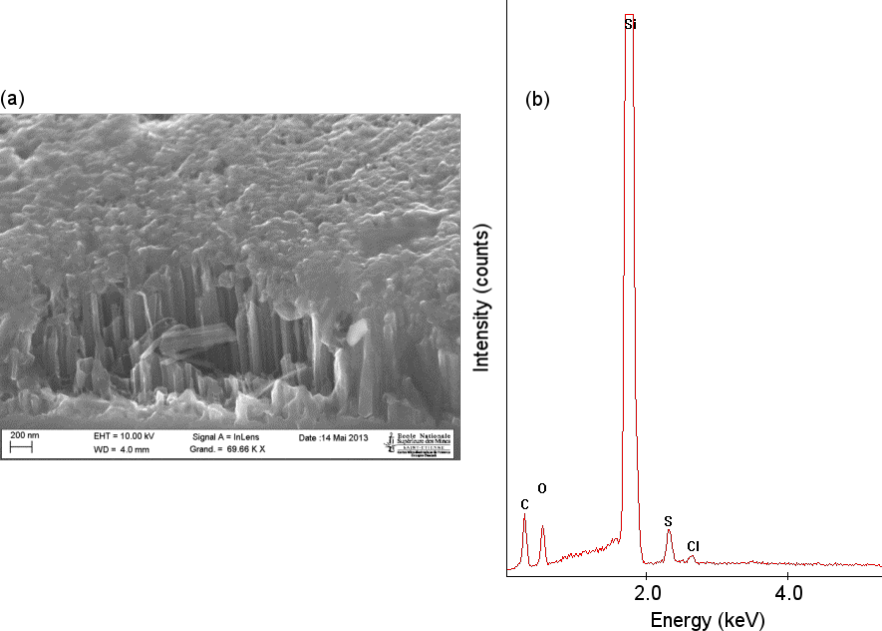
(a) SEM tilted-view of PEDOT covering the top of a SiNW array after 5 s of electrodeposition; (b) corresponding EDX spectrum.

SEM experiments and EDX analysis were then performed on a PEDOT deposit on SiNW substrate (Figure 9a,b). The SiNWs were fabricated using the EMACE method described in the Experimental section. The PEDOT was electrochemically deposited in a potentiostatic manner at a fixed 1.5 V potential for 5 s in a 10 mM EDOT ACN solution with 0.1 M LiClO_4_.

The SEM image in Figure 9a shows a damaged area which occurred during the cutting of the sample. A continuous film is formed on the top of silicon wires. The presence of S and C in the EDX spectra of Figure 9b proves that the film is primarily PEDOT. The space between the Si nanowires seems not to be filled by the polymer. This is caused by the competition between PEDOT polymerization on the tips and EDOT diffusion from solution to the inner space at the bottom. EDOT monomer that is more highly oxidized is produced in proximity of the tips of silicon wires. This is because the sharper morphology of the wire can lead to a much more intense electric field, resulting in an increase in polymer volume at the tips, and finally producing a mushroom-like morphology. With regards to the expanded polymer blocks of EDOT diffusion into the space, the 2D spread of PEDOT along the wire surface towards the bottom might also be prevented due to the lack of sufficient EDOT monomer.

### Effect of EDOT diffusion on PEDOT morphology

**Figure 10:**
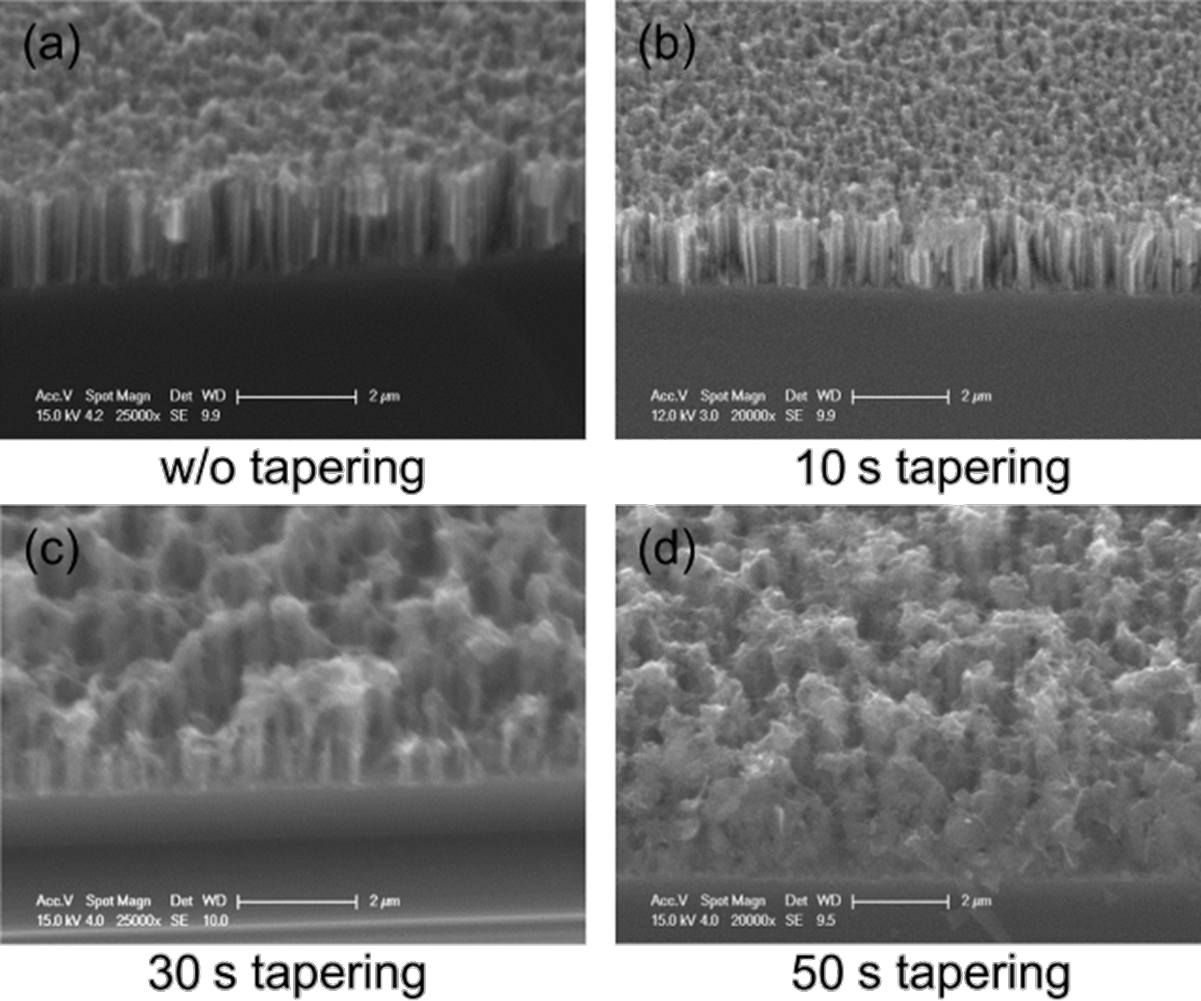
Tilted-view SEM images of SiNWs after PEDOT deposition at 1.5 V pulse deposition with a 10 mM EDOT + 0.1 M LiClO_4_ in acetonitrile solution (a) without tapering, (b) after 10 s tapering, (c) after 30 s tapering and (d) after 50 s tapering.

In order to achieve a conformal deposition, the previously discussed tapering method for the SiNWs was coupled with pulse deposition (5 cycles of 1 s on-time at 1.5 V and 10 s off-time at open circuit potential) in an attempt to force the EDOT oxidation along the entire SiNW. As described in the tapering process, the interspace volume between wires was changed by tuning the tapering durations to 0, 10, 30, and 50 s (see Figure 1). These tapered SiNWs samples were then dipped into the EDOT solution and the pulse method was performed to develop the polymer (see Figure 10a–d). Compared with the shorter tapering times (and therefore, a weaker diffusion effect) in Figure 10a and Figure 10b, the PEDOT films in Figure 10c and Figure 10d are thicker and PEDOT can be seen at the bottom and between wires. In contrast, for the samples presented in Figure 10a and Figure 10b, the space between the wires is very narrow; EDOT molecules cannot diffuse along the wires, and a mushroom-like morphology is obtained.

**Figure 11:**
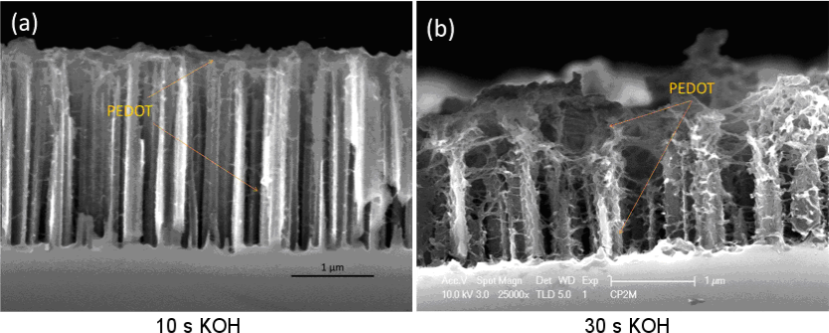
Cross-sectional view of HRSEM images of SiNWs/PEDOT sample after 10 s tapering (a) and 30 s tapering (b).

**Figure 12:**
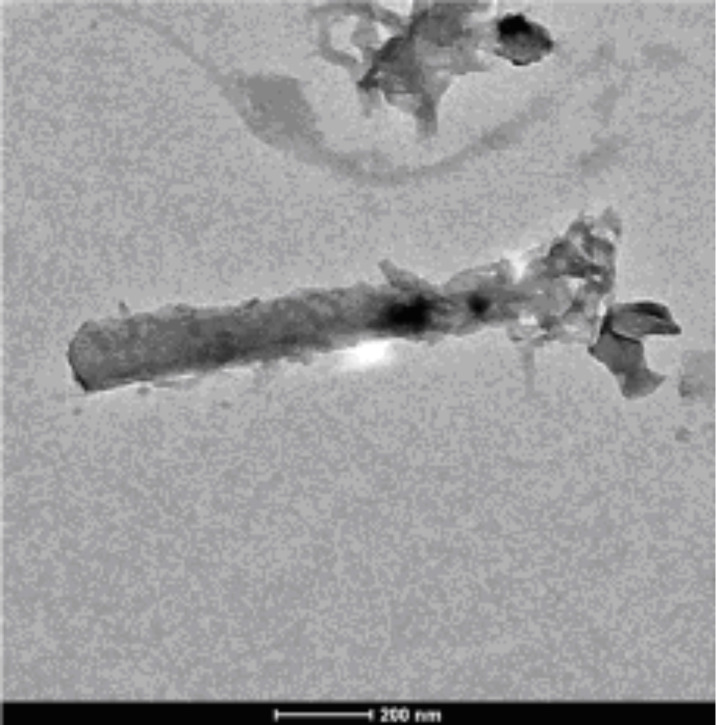
TEM image of a single SiNW/PEDOT after 30 s of tapering (corresponding to the sample in 11b).

These results are more evident in Figure 11, which shows high-resolution SEM images for two tapering durations (10 and 30 s). Figure 11a shows a cross section of the 10 s tapered sample, where the PEDOT is mainly concentrated on the top of the wires and only a small amount of PEDOT is formed at the bottom. This leads to an increased roughness of the tubes. However, Figure 11b presents a cross section of the 30 s tapered sample, where the tubes appear to be highly roughened due to the complete wrapping of the PEDOT from the top to the bottom. Moreover, the TEM image in Figure 12 of this 30 s tapered sample further demonstrates that the entire surface of a wire is wrapped with the polymer. The increased space between wires allows more EDOT to diffuse into the bottom space of wires during the electrodeposition and finally leads to a more conformal PEDOT covering layer.

**Figure 13:**
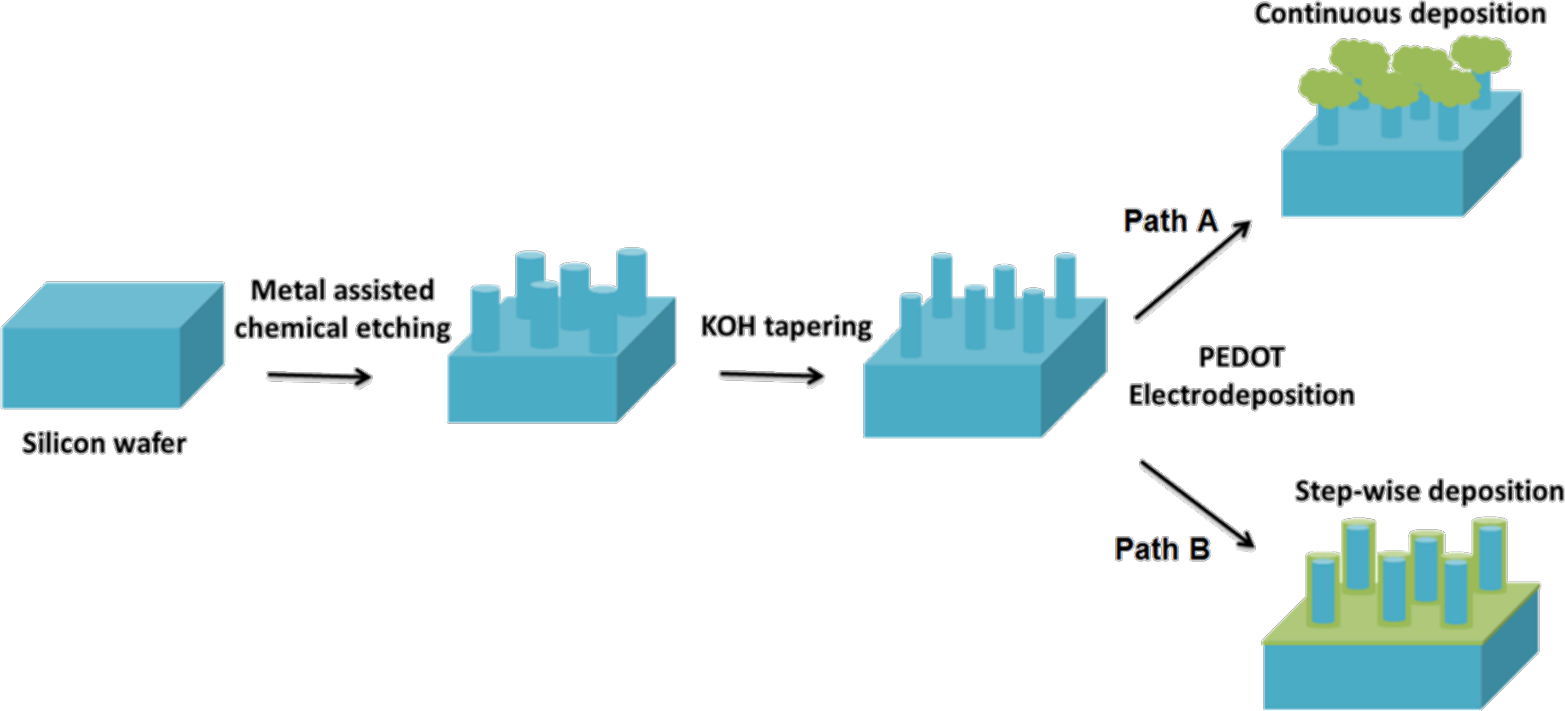
Schematic illustration of the processes resulting in PEDOT/SiNW hybrid structures. (a) Continuous electrodeposition leads to a mushroom-like morphology of PEDOT (green) on the tips of SiNW (blue), while (b) a step-wise deposition aids in the formation of a conformal PEDOT layer (green) surrounding each individual SiNW (blue).

The morphology of PEDOT on SiNWs is usually dependent on the synthesis conditions. Path A in Figure 13 identifies a continuous deposition that corresponds to CV or potentiostatic deposition experiments. Path B in Figure 13 corresponds to a step-wise deposition, which favors EDOT monomer diffusion into the space between the wires and thus results in a conformal layer.

### Effect of EDOT diffusion on diode quality

**Figure 14:**
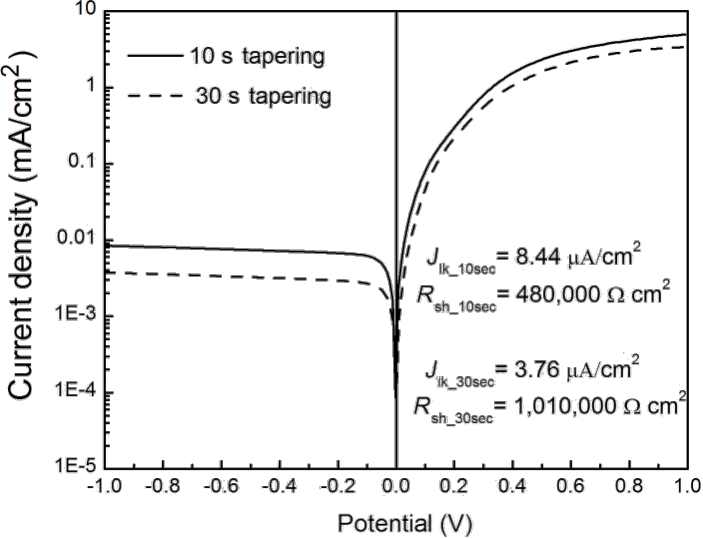
Current–potential characterization of the diodes with SiNW arrays tapered for 10 s (solid line) and 30 s (dashed line).

We further characterized the two PEDOT/SiNWs diodes with SiNWs tapered for 10 s and 30 s. As can be seen in Figure 14, both the 10 s and 30 s tapered devices show a distinct diode behavior with a constant reverse current–density curve in the negative voltage range. This is an indication that most of the shunt paths for charge transport at reverse voltages are blocked. An extremely large shunt resistance (*R*_sh_ > 1 M∙Ω∙cm^2^) and a very low leakage current density (*J*_lk_, on the order of μA/cm^2^) could be realized with this electrochemical method. The *R*_sh_ is three orders of magnitude higher than that of the reported diodes made by spin coating PEDOT onto SiNWs arrays [27]. This may be attributed to a much more conformal coating and much better adhesion of electrodeposited PEDOT on the Si nanowires. Indeed, it was found that the deposited PEDOT is difficult to remove from the Si surface with a taper because of the strong adhesion to Si.

The ultrahigh shunt resistance is directly related to the morphology of PEDOT surrounding the Si wire. In the current–potential characterization of a SiNWs/PEDOT diode in Figure 14, the *R*_sh_ and the *J*_lk_ for the 30 s sample are better than for the 10 s tapered diode. Since the primary shunt path can be formed by a top metal contact directly touching the n-doped Si through the PEDOT layer, a continuous PEDOT layer that separates n-doped Si from the top contact may prevent charge from tunneling through PEDOT. Therefore, the thicker layer formed after 30 s of tapering reduces the saturation current more than in the case of the 10 s tapered SiNW diode. We note that the forward current density for both diodes is relatively low: this could be due to the PEDOT conductivity, which is currently under study.

## Conclusion

In the present study, the preparation of a hybrid SiNW/PEDOT material using both chemical and electrochemical methods has been studied. The resulting structures based on silicon nanowires show interesting antireflective properties, with a reflection as low as 2% in the visible spectral range of 400–800 nm. PEDOT electrodeposition was successfully performed on illuminated, n-type SiNWs. The SEM images of the samples reveal that using a continuous deposition technique, the PEDOT layer covers primarily only the top of the Si wires due to the EDOT diffusion which affects the morphology of the polymer film. For a conformal, uniform deposition, pulse techniques were combined with etching of the SiNWs which allowed the monomer to better diffuse along the wires. The main advantage of this approach lies in the simplicity of both the SiNWs and PEDOT, both allowing for efficient and low-cost methods such as chemical etching and electrochemical deposition. To our knowledge, these two processes have never been combined for the production of such a hybrid material.

## Experimental

### SiNW etching and tapering

Before chemical etching, the Si wafers (phosphorus-doped, <100> oriented, resistivity 1–10 Ω∙cm, thickness 255–305 μm) were ultrasonically cleaned for 15 min in acetone and isopropanol. After several rinsing steps in deionized water, the wafers were immersed in piranha solution (H_2_SO_4_:H_2_O_2_ 3:1 v/v) and subsequently in deionized water for 15 min.

The EMACE technique is described in the literature [28] and is presented briefly here. During the Ag plating step (Step 1), the Si wafers were immersed into an aqueous solution of 4.8 M HF and 4 mM AgNO_3_ for 1 min. During the SiNW etching step (Step 2), the Ag-plated wafer was transferred to another solution of 0.3 M H_2_O_2_ and 4.8 M HF. The Ag network acts as a catalyst for the SiNWs etching, which was carried out for 2 min. The Ag catalyst was finally (Step 3) dissolved by immersion into 69% HNO_3_ for 30 min. The chemical reactions associated to each step are the following:

Step 1: 4Ag^+^ + Si + 6F^−^ → 4Ag + SiF_6_^2−^

Step 2: 2H_2_O_2_ + 4H^+^ + Si + 6F^−^ → 4H_2_O + SiF_6_^2−^

Step 3: 3Ag + NO_3_^−^ + 4H^+^ → 3Ag^+^ + NO + 2H_2_O

A tapering process was used to increase the EDOT diffusion by increasing the space between wires. The etched SiNWs were immersed into a KOH aqueous solution containing isopropyl alcohol (IPA) (31.2 g KOH + 25 mL IPA in 100 mL deionized water) for 10, 30 and 50 s. The addition of IPA was used to reduce the tapering rate [29].

### Core–shell structure realization

The PEDOT deposition was conducted in an electrochemical cell with a three-electrode configuration. The reference electrode was Ag/AgCl and the counter electrode was a platinum plate. All the potentials were indicated versus the Ag/AgCl reference electrode (*E*_Ag/AgCl_ = 0.192 V/SHE). A disc of vitreous carbon or the n-type SiNW array was set as the working electrode. For the latter, an illumination source consisting of a 150 W halogen lamp was used during the PEDOT polymerization to render the n-type Si substrate conductive in the anodic area. The deposition was controlled by a Solartron SI 1287 with a computer running CorrWare software.

A non-aqueous medium was preferred over the classical sodium polystyrene sulfonate (NaPSS) aqueous environment in order to avoid the important silicon oxidation during PEDOT deposition. An EDOT solution containing 10 mM EDOT and 0.1 M LiClO_4_ in anhydrous acetonitrile was used.

During the CV measurements, the potential scan was varied linearly in time in the direction from the cathodic to the anodic region, then the potential sweep was reversed to the beginning. This ramp in potential was repeated several times. The scan rate was 100 mV/s.

The deposit was first realized using a continuous deposition mode with a constant 1.5 V potential for 5 s. The pulse deposition technique was also used with the aim to improve the coverage of the nanowires with the polymer. The pulses consisted of 5 cycles of 1 s on-time at 1.5 V and 10 s off-time where the system was held at its open circuit potential. The total duration for the PEDOT deposition was the same (5 s) as that used for the continuous deposition technique.

After electrodeposition, the PEDOT on SiNW sample was rinsed with acetonitrile several times to remove excess EDOT monomer. The PEDOT/SiNWs sample was rinsed thoroughly with ethanol and then dried with an air stream.

### Characterization methods

The spectrophotometric measurements were performed with an integrating sphere provided by Sphereoptics. The SEM images and EDX spectroscopy were recorded with a Philips XL ESEM. The TEM observations were made with a TECNAÏ G20 instrument. Reflection FTIR spectra of PEDOT thin films were obtained using a VERTEX 70 spectrophotometer. Current*–*potential curves were measured using a Keithley 2400 instrument in a dark environment.
